# How Trend of Increasing Data Volume Affects the Energy Efficiency of 5G Networks

**DOI:** 10.3390/s22010255

**Published:** 2021-12-30

**Authors:** Josip Lorincz, Zonimir Klarin

**Affiliations:** 1 Faculty of Electrical Engineering, Mechanical Engineering and Naval Architecture (FESB), University of Split, R. Boskovica 32, 21000 Split, Croatia; 2 Polytechnic of Sibenik, Trg Andrije Hebranga 11, 22000 Sibenik, Croatia; zklarin@vus.hr

**Keywords:** green communications, green networking, 5G, radio access network, base station, energy-efficiency, metric, data, coverage, power, mobile network operator, wireless

## Abstract

As the rapid growth of mobile users and Internet-of-Everything devices will continue in the upcoming decade, more and more network capacity will be needed to accommodate such a constant increase in data volumes (DVs). To satisfy such a vast DV increase, the implementation of the fifth-generation (5G) and future sixth-generation (6G) mobile networks will be based on heterogeneous networks (HetNets) composed of macro base stations (BSs) dedicated to ensuring basic signal coverage and capacity, and small BSs dedicated to satisfying capacity for increased DVs at locations of traffic hotspots. An approach that can accommodate constantly increasing DVs is based on adding additional capacity in the network through the deployment of new BSs as DV increases. Such an approach represents an implementation challenge to mobile network operators (MNOs), which is reflected in the increased power consumption of the radio access part of the mobile network and degradation of network energy efficiency (EE). In this study, the impact of the expected increase of DVs through the 2020s on the EE of the 5G radio access network (RAN) was analyzed by using standardized data and coverage EE metrics. An analysis was performed for five different macro and small 5G BS implementation and operation scenarios and for rural, urban, dense-urban and indoor-hotspot device density classes (areas). The results of analyses reveal a strong influence of increasing DV trends on standardized data and coverage EE metrics of 5G HetNets. For every device density class characterized with increased DVs, we here elaborate on the process of achieving the best and worse combination of data and coverage EE metrics for each of the analyzed 5G BSs deployment and operation approaches. This elaboration is further extended on the analyses of the impact of 5G RAN instant power consumption and 5G RAN yearly energy consumption on values of standardized EE metrics. The presented analyses can serve as a reference in the selection of the most appropriate 5G BS deployment and operation approach, which will simultaneously ensure the transfer of permanently increasing DVs in a specific device density class and the highest possible levels of data and coverage EE metrics.

## 1. Introduction

The trend of constantly increasing the number of mobile users consequently leads to an increase in traffic data volumes (DVs). It is projected that global mobile data traffic will exceed 300 EB per month in 2026 [1]. The overall number of Massive Machine-Type Communications (mMTC) and non-mMTC connected devices in the fifth-generation (5G) networks will increase from 165.6 million in 2020 to 3.256 billion in 2030, with a compound annual growth rate (CAGR) of 35% [2]. All 5G connected devices will, by 2030, account for 13% of the overall number of connected devices (25.4 billion) worldwide [2]. Although the fourth-generation (4G) mobile networks will remain the dominant mobile access technology by 2025, the market uptake of the 5G mobile network is faster than that of 4G, and 5G is expected to overtake 4G in the second half of the 2020s.

As the next step of wireless network evolution, the 5G network will enable new applications and services. Most of them will request greater network capacity and data rates. For some use cases, these requirements are accompanied by a demand for ultralow latencies, ultra-high reliability or exceptional availability [3]. Additionally, the rapid growth in the number of connected devices is expected to sustain in the future. This growth will mainly be due to emerging applications, such as ultrahigh-definition (UHD) video, augmented and virtual reality, fixed wireless access, intelligent transportation, remote healthcare, industrial automation, Internet of Everything (IoE) networks, smart agriculture, smart cities, etc. Such applications will be characterized by an enormous number of devices that will generate a huge amount of traffic DVs, which will contribute to the continued global growth of DVs during the 2020s.

As the existing microwave spectrum becomes severely congested, the 5G network, as the first among all previous mobile network generations, enables communication in a frequency spectrum above 6 GHz and in millimeter-wave (mmWave) spectrums (above 30 GHz). A larger amount of available bandwidth in above 6 GHz bands enables multi-gigabit data rates through wider wireless channels. Wider bands also enable higher channel reuse, which further allows denser implementation of macro and small base stations (BSs) having significantly higher capacities than those in previous generations of mobile networks.

However, the drawback of transmission in above 6 GHz bands is in high propagation losses and susceptibility to blockages of short-wavelength signals, which reduces the operational coverage of such communication systems. As a consequence, the 5G radio access network (RAN) deployment is envisioned as a heterogeneous network (HetNet). Such HetNets consist of a small number of 5G macro BSs and a huge number of 5G small BSs. Macro BSs support indoor and outdoor basic signal coverage and capacity demands for wider geographic areas, while accompanying small BSs (micro, pico or femto BSs) accommodate most of the capacity demands for both indoor and outdoor geographic areas characterized as hotspots with high traffic DVs.

Deployment of such HetNets composed of densely allocated macro and small BSs, accompanied with the need for satisfying transfer of constantly increasing traffic DVs, will have a non-negligible impact on energy consumption of radio access part of 5G and the future sixth-generation (6G) mobile networks. Therefore, in this study, the influence of the increasing traffic DVs on the energy efficiency of 5G networks was analyzed. The analyses were performed for energy-efficiency (EE) metrics standardized by the European Telecommunications Standards Institute (ETSI). Moreover, the DVs used for analyses are simulated DVs, which amount to specific device density classes (rural, urban, dense urban and indoor hotspot); they were selected according to the maximal expected number of 5G devices per square kilometer of each device density class defined in ETSI standards. Analyzed device density classes represent expected device densities which average uplink (UL) and downlink (DL) throughputs prescribed by ETSI standards and contribute to the total expected DV of one of four specific device density classes. These device density classes represent typical operating environments served by corresponding 5G HetNet deployments. Performed analyses in this study highlight the impact of such an increase in DVs on future trends of 5G network EE metrics, with respect to different deployment and operating HetNet scenarios and device density classes.

The rest of the paper is organized as follows: In Section 2, an overview of energy requirements of 5G networks is been presented. In Section 3, standardized metrics used for evaluating 5G network EE are elaborated. The types of different device density classes used in the analyses are explained in Section 4. Modeling of DVs’ increase used for analyses of the impact of increasing DVs on EE of 5G networks is described in Section 5. The 5G radio access network (RAN) deployment and operation approaches and their performance in terms of EE are presented in Section 6. Section 7 analyses the results, which indicate how continuous increase in DVs impacts the values of EE metrics, instant power consumption and yearly energy consumption of the 5G network. In Section 8, a discussion on performed analyses and obtained results is performed. Finally, conclusions related to the analyses presented in the paper are given in Section 9.

## 2. Energy Requirements of 5G Networks

The deployment of a large number of new 5G macro and especially small BSs dedicated to satisfying trends of ascending DVs will have a significant impact in terms of increased energy requirements related to the power supply of BSs in RANs. Even the energy consumption (EC) reduction expected through the removal of equipment characteristic for some older network generations (e.g., second and especially third), due to obsolescence, will be diminished with the need for installing a vast number of 5G BSs. Therefore, the expected future increase of DVs in mobile access networks will undoubtedly affect the realization of 5G networks according to the goals of the green communications and networking paradigm. Green communications and networking is an emerging strategy dedicated to the development of novel energy-efficient solutions, which will minimize the usage of network resources for reducing network energy consumption whenever possible.

The design of previous cellular network generations was dominantly focused on improving network performance through maximizing throughput and spectral efficiency at the expense of EC. The EE improvements contributing to the realization of the green communications paradigm in pre-5G cellular or local wireless networks were mainly dedicated to solutions related to BSs transmit power and activity adjustments according to dynamic variations of DVs [4,5]. Moreover, green communication solutions related to the supplying of BSs site with renewable energy sources, which have been capacitated according to the daily and monthly traffic DV variations on BS site, have been envisioned as a possible solution to improve network energy efficiency [6].

Although hardware components of 5G BSs are more energy-efficient than those of previous generations of BSs (2G, 3G and 4G), the amount of EC of 5G radio access networks is expected to increase due to the need for fulfilling new 5G requirements, such as accommodation of vast DVs, ultrahigh data rates and/or ultralow latencies needed for ensuring some new services. This increase in the EC presents not just an economic issue reflected in an increase of operational costs for mobile network operators (MNOs), but also in an environmental issue due to increased carbon emissions and strategic issue due to alienation from the main goals of green communications paradigm. Therefore, ensuring the energy-efficient operation of RAN in 5G networks has become one of the major challenges analyzed in the literature [7,8].

This challenge is further contributed to by the fact that MNOs must adapt the number of BSs and corresponding capacities according to the expected number of users and their DVs. Classification of device density classes (areas) in 5G networks performed by ETSI in References [9,10] indicates that the number of users and corresponding DVs can significantly vary per specific device density class (Table 1).

Moreover, due to the global population growth, the advent of new and more capacity-demanding applications, the proliferation of the Internet of Everything (IoE) concept and the increase in the number of devices per user, the DV increase in any device density class will have continuous progress throughout the 2020s. Following this progress in terms of satisfying the capacity demand of each device density class raises challenges on MNOs in terms of keeping 5G RAN deployment and operation as energy efficient as possible. These challenges can be precisely expressed in changes of EE metrics for specific device density classes of 5G networks. In our preliminary work, the impact of the increasing number of active users in 5G networks on network EE metrics was analyzed [11]. However, in mobile access networks, the increasing trend of changes in user densities is not the same as the increasing trend of changes in data volumes (DVs). Therefore, analyses dedicated to the explanation of how the expected increasing trend of DVs during the 2020s impacts EE of 5G RAN have not been analyzed yet.

Satisfying this increasing trend of DVs can be performed in 5G RANs through the exploitation of different small and macro BSs implementation and operation scenarios. They can differ in the number of used BSs and corresponding ECs, which ultimately influence on 5G network EE. The EE in the 5G RAN can be validated through recently standardized metrics which enable consistent evaluation of RAN EE in different device density classes. Hence, the impact of the increased DVs on EE metrics of 5G RANs in versatile device density classes was analyzed in this work for the first time. The analyses give an explanation of how the ascending trend of DVs impacts distinct EE metrics. Moreover, the analyses for the first time clarify the impact of increased DVs on instant power and yearly energy consumption of the 5G RAN.

## 3. Metrics for Evaluating 5G Network Energy Efficiency

Recently, the main standardization organizations, such as the 3rd Generation Partnership Project (3GPP), the ETSI and the International Telecommunication Union-Telecommunications Standardization Sector (ITU-T), accepted two types of the EE metrics as the key performance indicators (KPIs) for expressing the EE of mobile networks (MNs).

The first metric represents the data EE metric of MN expressed in bit/J, and it is defined as the amount of data transmitted per Joule of energy. For known mobile network area, *A*, data *EE* is expressed as follows:

(1) $$EE_{DA}=\frac{DV_{A}}{EC_{A}}\;\left[\mathrm{bit}/J\right]$$

According to Equation (1), the data EE metric ($EE_{DA}$) is calculated as the ratio between the overall DV ($DV_{A}$) transferred by the analyzed MN area and energy ($EC_{A}$) needed by MN equipment installed in this area for transferring these DVs.

Another standardized EE metric expressed in m^2^/J represents the coverage EE of the MN area, and it is defined as the unit area which can be covered with the 5G wireless signal, using Joule of consumed energy. For known mobile network area, *A*, the coverage EE is expressed as follows:

(2) $$EE_{CA}=\frac{S_{A}}{EC_{A}}\;\left[m^{2}/J\right]$$

Based on Equation (2), the coverage EE metric ($EE_{CA}$) is calculated as the ratio between the overall size of the area ($S_{A}$) covered with the wireless signal of the analyzed MN and the total energy ($EC_{A}$) consumed by the equipment allocated in the analyzed MN area. The total EC ($EC_{A}$) of the analyzed MN area can include the sum of the energies consumed by the RAN equipment (BSs), transmission equipment (e.g., wired or wireless backhauling equipment and radio controllers) and all ancillary equipment (e.g., air-conditioning, power backup, etc.).

These two standardized metrics are used to effectively analyze the deployment and operation performance of MN in terms of EE. They can be used for the assessment of MN EE performance in the case of various deployment and operation strategies of different BS types and device density classes [1,2]. As different deployment and operation strategies for a new generation of MN can be implemented in practice, the EE assessment of mobile networks by using standardized metrics becomes crucial for MNOs when assessing approaches related to the deployment and operation of new generations of mobile networks.

## 4. Device Density Classes for Assessment of Energy Efficiency

**Figure 1:**
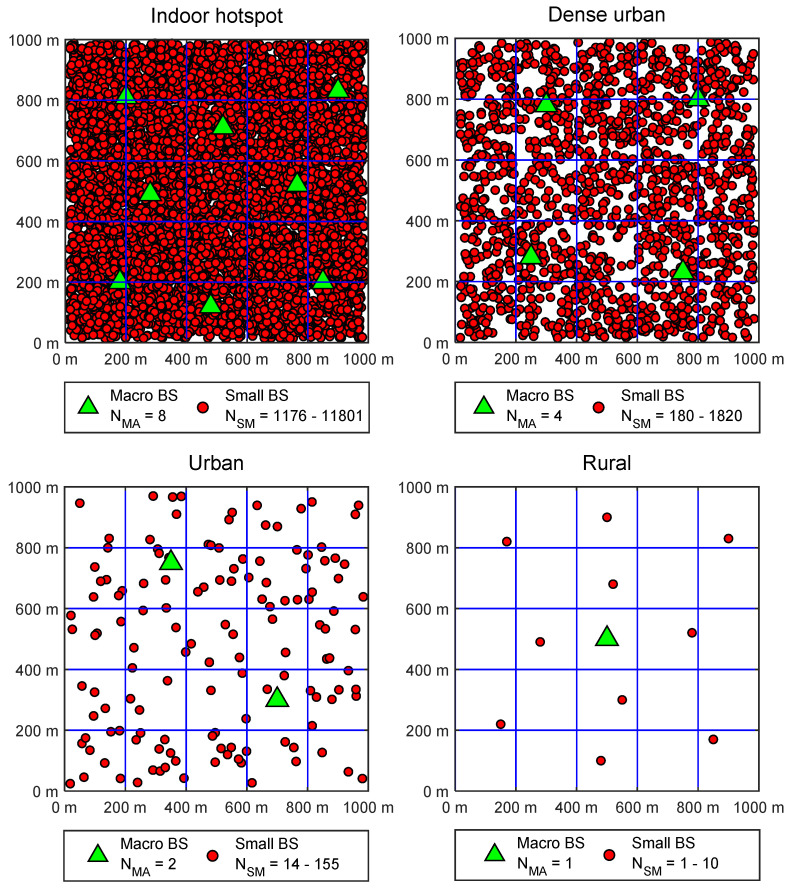
Visualization of analyzed 5G HetNet deployments for different device density classes with the maximal number of installed small and macro BSs per square kilometer area.

The method for EE evaluation of 5G RAN was presented in ETSI standard (ES) 203 228 [9]. According to this method, when the measurement of parameters needed for EE assessment of complete MN on the large scale is not feasible, the total network can be divided into a set of smaller sub-networks that are classified according to device densities of a specific sub-network. For EE assessment of 5G RAN presented in this work, analysis was performed by using sub-networks categorized by device density classes for an area of one square kilometer (1 km^2^). Figure 1 visualizes analyzed 5G HetNet deployments for different device density classes with the maximal number of installed small and macro BSs per square kilometer area.

The impact of DVs on different EE metrics can be analyzed for different device density classes which are defined in ETSI standards [9,10]. They can range from the rarely populated rural device density class having up to hundreds of users per square kilometer (km^2^) to the urban, dense-urban and densely populated indoor-hotspot device density classes, having up to a few hundreds of thousands of users per km^2^. The maximal densities of user devices in specific device density classes for the analyzed area of one square kilometer are presented in Table 1. Hence, the device density classes presented in Table 1 are classified depending on the maximal user density of the square kilometer area (users/km^2^).

As an example, Figure 1 visualizes analyzed device density classes of the 5G RAN deployment in terms of the number of allocated macro and small BSs. Analyzed HetNet 5G RAN deployment architectures are, for each of the device density classes, composed of a few macro BS(s) and a large number of small BSs (Figure 1). Figure 1 presents a maximal number of macro BSs allocated in each device density class. In the analyses, it is assumed that the number of macro BSs is constant during the 2020s and corresponds to the number of BSs allocated during the initial deployment of the 5G network. This approach to deployments of macro BSs is frequent in practice, since MNOs deploy macro 5G BSs to ensure initial coverage and capacity demands of a specific area. In areas expecting larger DVs, a larger number of macro BSs will be initially deployed, and this explains the differences in the maximal number of macro BSs deployed in different device density classes in Figure 1. Moreover, a number of small BSs will be deployed according to different deployment and operation approaches described and analyzed in further sections. Since the deployment of the number of small 5G BSs in some analyzed areas depends on different MNO deployment and operation approaches, in Figure 1, the span of small BSs that can be deployed is indicated in the range from minimal to the maximal number of small BSs that are considered for analyzes in different approaches. Therefore, Figure 1 illustrates basic MNO principles of RAN deployment analyzed in this work, according to which a few macro BSs are deployed for ensuring basic signal coverage and capacity of the analyzed area and a larger number of deployed small BSs are dedicated to satisfying capacity demands at locations of traffic hotspots.

## 5. Modeling of DVs Increase in 5G Networks

A continuous increase in DVs will ultimately affect the 5G RAN layout in terms of the needed number of BSs that must be deployed in a specific device density class (area). According to Reference [12], for each device density class, different requirements in terms of expected average uplink (UL) and downlink (DL) data rates per active user have been defined by the ETSI standard [1,2] (Table 1). Defined average UL/DL data rates assume that less populated areas (e.g., rural) require lower average throughputs and ultra-densely populated areas (e.g., indoor hotspot) require average throughputs that are significantly higher.

In this work, the DV increment was simulated for each year in the 2020s. The simulation is based on the total expected DV increase during the 2020s. This increase in DV is calculated for each year in the 2020s, based on the maximal expected number of 5G devices per square kilometer in each device density class prescribed by the ETSI standards (Table 1) [1].

According to values of UL and DL average throughputs and maximal user densities defined in References [9,10] (Table 1), the overall impact of trend in DV increase on EE metrics of specific device density classes was modeled. For analyses in this paper, this impact was modeled by scaling the DV in each device density class in the range from 10% to 100% of a total average UL and DL DV. The total UL and DL DV were obtained for the maximal number of user devices per square kilometer area of every device density class prescribed by the ETSI. More specifically, the 10% of maximal expected DV in 2030 was used for representing DV in 2021, and for each subsequent year in the 2020s, the increase in DVs is assumed to be incremented by 10% up to 2030. For the year 2030, the maximal average DV defined by total UL and DL traffic of the maximal number of user devices per square kilometer prescribed by ETSI in References [9,10] is used in the analyses (Table 1). Through modeled increases in DVs for each device density class, it is shown how an increasing trend of DVs affects the data and coverage EE of the specific device density class.

Based on the demands for the transfer of DVs in each device density class, a different number of installed macro (N_MA_) and small (N_SM_) BSs were allocated in the analyzed area of the square kilometer (Figure 1). An increase in DVs ranging from 10% to 100% of a total maximal DV in each device density class was accommodated through the deployment of an appropriate number of small BSs. For that reason, ranges of the number of small BSs in specific device density classes are also presented in Figure 1.

The overall number of installed macro (N_MA_) and small (N_SM_) BSs presented in Figure 1 depends on the increase in DVs of each device density class toward the maximal expected DV presented in Table 1. The installed number of macro and especially micro BSs in a specific device density class presented in Figure 1 is related to the overall capacity that active BSs in RAN must have for ensuring the transfer of expected DVs. The capacity of each BS type is defined based on operating parameters of macro and small BSs, as presented in Table 2 [13]. They are characteristic for the typical contemporary 5G BSs market models, and, based on them, the overall RAN capacity in terms of a minimal number of the macro and small BSs needed for the transfer of expected DVs in each device density class was allocated. Allocation of 5G BSs is performed in accordance with an increase of DV in the specific device density class. As the demand for transfer of higher DVs increases and the current capacity of installed 5G small BSs reaches maximal exploitation, the new small BSs are deployed in the network on positions of traffic hotspots (Figure 1). This concept of adding new BSs in the network simulates some of the analyzed approaches dedicated to the realization of future 5G networks, which will be realized through the gradual deployment of new small 5G BSs on positions of traffic hotspots.

According to Table 1, the percentage share of the transfer of DVs performed by macro BSs is lower for device density classes having higher DVs (user densities), and vice versa. Hence, in rarely populated rural device density classes, most of the DVs will be transferred over macro BSs. However, in the indoor-hotspot device density classes characterized by the necessity of transmitting huge DVs, macro BSs will transfer minor DVs, while the remaining DVs will be transferred by small BSs. Moreover, with the increase of DVs in specific device density classes, the involvement of macro BSs in the transfer of DVs will decrease on the account of small BS.

## 6. Simulation of 5G RAN Deployment and Operation Approaches

MNOs can exploit different approaches for the initial deployment of new BSs in 5G RANs. However, the selection of the best 5G network deployment approach in terms of keeping network EE at optimal levels is still an open question to MNOs. As in the case of previous generations of the cellular networks, implementation of the 5G network can last for years until full deployment in terms of needed capacity and coverage on the national level of complete countries will be achieved. According to the prediction in Reference [1], it is expected that 60% of the world population will be covered with 5G signal by 2026. By that period, installed 5G RAN resources will also need to accommodate increased DVs, which will be significantly higher than the ones at the first years of 2020s.

However, different deployment approaches of BSs in 5G RANs can be realized through versatile deployment and RRM techniques. They can have a different long-term impacts on the EC of the RAN. Selecting an appropriate deployment and RRM approach in terms of EE can contribute to the reduction of network EC and consequently to the improvement of the EE metrics. Hence, further analyses reveal how different 5G RAN deployment approaches and continuous increase of DVs in the 2020s influence the 5G network EE metrics. As the largest share of the 5G network EC is related to the EC of BSs in 5G RAN [6], further analyses are performed only for deployment and RRM approaches related to 5G BSs only.

Due to the known fact that many active BSs are lightly loaded for most of their operating time, during which active BSs consume energy [3], analyzed deployment and RRM approaches exploit this fact for potential energy savings. More specifically, an operating strategy based on the switching of small BSs in sleep operation mode during the idle traffic periods is considered in the analyses [14,15]. This operating strategy is considered, since the radio resource scaling (in terms of BS transmit (Tx) power levels, number of active transceivers, number of active subcarriers, etc.) according to dynamics of the user’s activity has been proven as an approach that contributes to enhancement of the HetNets EE [4,5,16]. Such concepts dedicated to improving BSs EE are included in some contemporary types of the 5G small BSs [17], and even more advanced concepts will be implemented in the future releases of 5G BSs.

Three different 5G BSs modes of operation are analyzed in the paper. In Table 2, instant average power consumption of those operation modes, which can be active, sleep and Tx power scaling, are presented. The power consumption of 5G BSs in active, sleep and Tx power scaling modes were selected for analyses based on Reference [3]. The selected BSs power consumption and configuration parameters presented in Table 2 are inherent to the preliminary models of 5G small and macro BSs. The power consumption of BS working in active mode represents the average power consumption of BS for which operating capacities are fully exploited at the highest Tx powers [3]. The average EC of the small BSs in sleep mode is estimated to be 10% of the average EC characteristic for small BS operating in full active mode (Table 2). Due to the necessity of ensuring constant coverage of the area with 5G signal, the possibility that macro BSs can be in sleep mode is not considered in the analyses (Table 2). The average power consumption of BSs in Tx power scaling mode is assumed to be 80% of the average power consumption in active mode (Table 2). The Tx power scaling mode is analyzed, since it is confirmed in References [4,5] that scaling of the Tx power of BSs according to the space and time DV variations can additionally give a contribution to the enhancement of the EE of a mobile access networks. Hence, a very conservative assumption related to the reduction of average instant BSs power consumption for 20% on a daily basis in comparison with a power consumption of small BSs constantly operating in the active state at the highest Tx power is used in this analysis.

Another MN characteristic that is used in the presented analyses is the fact that the deployment of BSs in cellular RANs is based on ensuring the projected capacity needs for satisfying peak network traffic volumes during a BS exploitation period of approximately 10 years since initial BSs deployment. Besides the fact that such an implementation imposes large initial capital investments for MNO, it also imposes increased operational costs in terms of large monthly energy bills paid by MNO for operating RAN with significantly larger capacities than those needed during most of the 5G RAN equipment lifetime. These larger 5G RAN energy costs consequently result in reduced 5G network EE, which can be improved if an appropriate 5G BSs deployment and operation approach is exploited. Hence, finding an optimal deployment and RRM approach from the EE perspective represents a crucial challenge to MNOs.

### 6.1. Types of 5G RAN Deployment Approaches

As previously emphasized, a rapid increase in mobile users and the high-throughput requirements of future applications will contribute to the constant increase of the DVs in 5G HetNets during the upcoming decade. Therefore, the analyzed BS deployment approaches simulate this growth of DVs for the 10-year period starting with the year 2021 in every device density class (Table 1). In Table 3, the main characteristics of the analyzed network deployment and operation approaches are presented. The analysis takes into account broadly accepted MNOs practice based on the initial deployment of the fixed number of macro 5G BSs for each deployment approach in every device density class (Figure 1). The number of installed macro BSs corresponds to the number of BSs required to provide a minimal level of signal coverage and capacity requirements for the transfer of expected DVs in a specific device density class (Figure 1).

The number of installed small BSs and corresponding RRM principles differs among deployment approaches (Figure 1), and this constitutes the main difference among the analyzed deployment approaches (Table 3). In order to model various BSs deployment and RRM approaches and compare their impact on the standardized EE metrics, five different types of deployment and operation approaches were selected for analysis.

#### 6.1.1. Approach 1—Variable Number of Small BSs in Active Mode

Deployment Approach 1 is characterized by the variable number of small BSs in active mode during their operational period (Table 3). This approach is based on 5G HetNet deployment strategy in which the number of installed small BSs is continuously increased over time. This increase is based on satisfying the demand for transfer of increasing DV traffic in specific locations of every device density class. This approach does not include any radio resource management (RRM) technique for improving BSs EE in periods when BSs are active (Table 3). Hence, those macro and small BSs that are installed (Figure 1) are constantly active with maximal EC during the entire working period. Their instant power consumption corresponds to the average instant power consumption of BSs working constantly in active mode (Table 2).

#### 6.1.2. Approach 2—Maximal Number of Small BSs in Sleep Mode

This deployment approach is characterized by the possibility of having a maximal number of small BSs in sleep mode during RAN operation (Table 3). In this approach, all small BSs needed for accommodating expected DVs in the upcoming period of 10 years are initially installed in the RAN by MNO. This approach exploits the small BSs sleep mode strategy, which enables the preservation of energy through putting small BSs in a sleep operation mode during the periods lacking the data needed to be transferred by those BSs. When the demand for capacity exceeds the available capacity of the currently active macro and small BSs, the required number of small BS(s) that are in sleep mode are activated. Moreover, this approach does not assume the implementation of any RRM method for improving EE of macro BS(s).

#### 6.1.3. Approach 3—All BSs Constantly in Active Mode

Deployment Approach 3 demonstrates the traditional approach to the deployment of BSs in which all (macro and small) BSs are constantly in active mode (Table 3). As in the case of deployment Approach 2, all small BSs needed for the accommodation of expected DVs in the upcoming 10-year exploitation period of 5G HetNet are initially installed in the RAN by MNO. The installed BSs operate without any adjustment of radio resources in accordance with time and space variations of DVs; thus, they consume constantly maximal energy. This approach characterizes the constant power consumption of small and macro BSs that corresponds to the average power consumption of BSs working in active mode (Table 2). This approach, which is traditionally exploited for the deployment of pre-5G BSs, is considered for comparison purposes with other more advanced BSs deployment and deployment approaches.

#### 6.1.4. Approaches 4 and 5—Variable Number of Small/All BSs in Tx Power Scaling Mode

Deployment Approaches 4 and 5 correspond to deployment Approach 1 in terms of the deployment of small BSs according to a gradual increase of DV over time (Table 3). In these two approaches, a BSs Tx power scaling according to time and space variations of DV is exploited, since it is proven that such a technique can additionally preserve the EC of the RAN. The Tx power scaling technique used for the purpose of the EE assessment in this analysis follows a conservative approach, assuming that implementation of such technique reduces EC of the BS for 20% of the EC which BSs have in case of transmitting at the highest Tx power (Table 2). In the case of deployment Approach 4, the Tx power scaling is applied only to the small BSs, while, in the case of deployment Approach 5, the Tx power scaling mode is applied to both small and macro BSs (Table 3).

## 7. Results on the Impact of Increasing DVs on 5G Network EE

**Figure 2:**
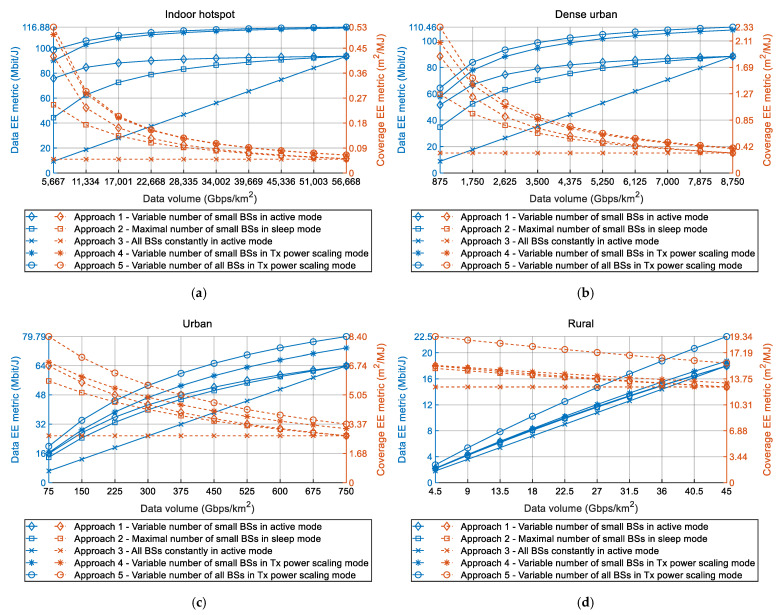
Impact of DV increase on data and coverage EE metrics for each year during the 2020s for (**a**) indoor-hotspot, (**b**) dense-urban, (**c**) urban and (**d**) rural device density class.

The impacts of the increase in DVs on data and coverage EE metrics of 5G RAN for different device density classes and five deployment approaches are shown in Figure 2a–d. Figure 2a–d indicated estimated DVs for each year during the 2020s, with 2030 as the last year for which DV estimation was performed. According to the presented simulation results, the increase in DVs has a significant impact on data and coverage EE metrics of 5G RANs. This impact is visible for every device density class and deployment approach. In the case of all deployment approaches and for every analyzed device density class, the data EE metric increases with the increase of DV that must be transferred in the 5G network (Figure 2a–d). Thus, an increase in DVs has a positive impact on data EE metrics of 5G networks. This implies that a higher amount of DVs can be transferred per Joule of energy consumed by the network BSs of the same area size. Therefore, the global trend of the constant increase of DVs in 5G networks will result in the improvement of the data EE metric. This improvement is a consequence of the fact that higher amounts of data will be transferred for the same unit of energy consumption of the BSs in the 5G RAN.

On the other hand, for every device density class and most of the deployment approaches, the coverage EE metric decreases as the DV of the analyzed square kilometer area increases (Figure 2a–d). This decrease is not perceived only for deployment Approach 3, which presents the traditional RAN implementation approach lacking any deployment or operational mechanisms dedicated to optimizing BSs energy consumption. In the case of other deployment approaches (Approaches 1, 2, 4 and 5), an increase in DVs has a negative influence on coverage EE metrics. This implies that ensuring the transfer of higher DVs over the same area requires more energy which will be consumed by the network elements (BSs) allocated in this area. Therefore, the global trend of the constant increase of DVs in 5G networks will result in the degradation of the coverage EE metric of the radio access part of the network. This decrease is a consequence of the fact that transferring higher amounts of data in a specific device density class demands an increase in the energy consumption of the BSs in the 5G RAN.

According to Figure 2a–d, an increase in DVs causes the opposite trends in changes of data and coverage EE metrics, where an increase in the data EE metric is followed by a decrease in the coverage EE metric. This is not the case only with deployment Approach 3, due to the above-explained reasons. Hence, there is no optimal 5G BSs deployment approach that can simultaneously contribute to the improvement of both standardized EE metrics. Favoring the data EE metric in terms of transferring higher amounts of DVs per unit of consumed energy will be on the cost of the degradation of coverage EE metric, and vice versa. However, to have as much energy-efficient 5G RAN as possible, both EE metrics must be simultaneously optimized in terms of obtaining network deployment and operation, which will have the highest possible data and coverage EE metric. This makes the realization of 5G HetNets in terms of satisfying both standardized EE metrics particularly challenging for MNOs. This challenge arises from the fact that there is no optimal DV around which MNOs should keep the traffic intensity in a specific device density class, for which both EE metrics will have the best possible values.

### 7.1. Impact of Increasing DVs on the Power Consumption of 5G Network

**Figure 3:**
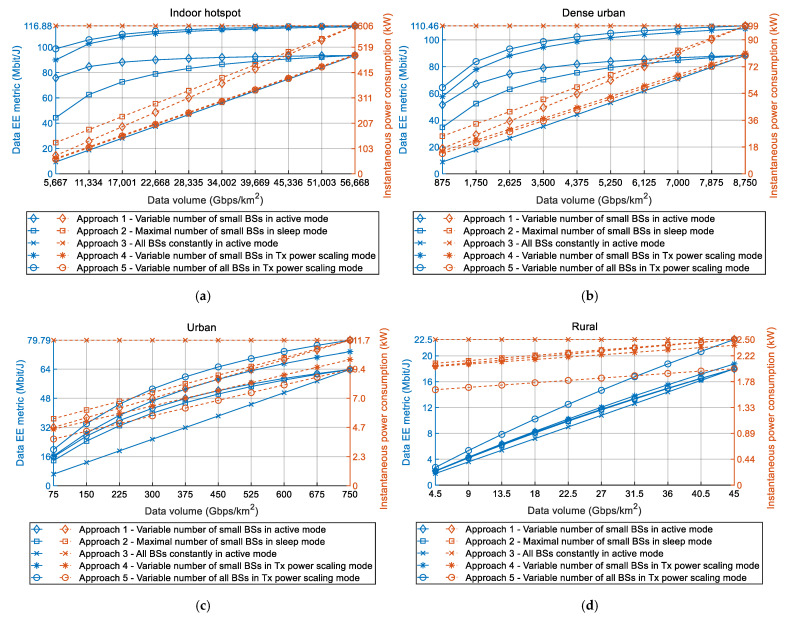
Impact of DV increase on data EE metrics and instant 5G network power consumption during the 2020s for (**a**) indoor-hotspot, (**b**) dense-urban, (**c**) urban and (**d**) rural device density class.

Since increasing DVs impact both EE metrics of 5G networks, an increase in DVs will also have an influence on instant 5G network power consumption. The instant network power consumption represents the total average instant power consumption of all (small and macro) BSs located in the analyzed device density class that are in an active and operating state (Table 2). Obtained results presenting the impact of DV increase on data EE metrics and instant 5G network power consumption for indoor hotspot, dense urban, urban and rural device density classes are shown in Figure 3a–d, respectively. Results presented in each figure have been obtained for all of the five different deployment and operation approaches and for DVs characteristic for every year in 2020s.

According to Figure 3a–d, for analyzed Approaches 1, 2, 4 and 5, an increase in DV of each device density class will impose an increase in the instant 5G network power consumption. This is due to the fact that transferring larger DVs requires more network resources in terms of activating an additional number of BSs, exploiting more capacity of active BSs (in terms of transceivers, channels, subcarriers and multiplexing slots) and transmitting at higher Tx power levels. This consequently results in higher instant power consumption of individual BSs, which jointly contributes to an increasing trend of total instant network power consumption in every device density class. The only exception from this power consumption trend is the instant power consumption of Approach 3 (Figure 3a–d). Since this approach lacks any adaptation of BSs deployment dynamics and operation activity according to DV variations, instant power consumption of the network will be constantly at maximal levels (Figure 3a–d). In comparison with other deployment and operation approaches, this results in the worse data EE metric of Approach 3.

Therefore, for any of the analyzed approaches (Approaches 1, 2, 4 and 5) which exploit adaptation of BSs deployment dynamics and operation activity according to increasing DV trend, this DV increase will have a negative impact in terms of increasing the instant power consumption of the 5G network. This negative impact is not translated to data EE metrics, which will increase with the increase of DV in all analyzed device density classes (Figure 3a–d). This is the consequence of the fact according to which instant power consumption and DV have different increasing rates. Although instant 5G network power consumption and, therefore, overall network energy consumption increase during the analyzed period of 10 years, the overall DV during this period also increases with higher rates, which in total contributes to the increase of data EE metrics calculated based on the Equation (1).

### 7.2. Impact of Increasing DVs on the Energy Consumption of 5G Network

**Figure 4:**
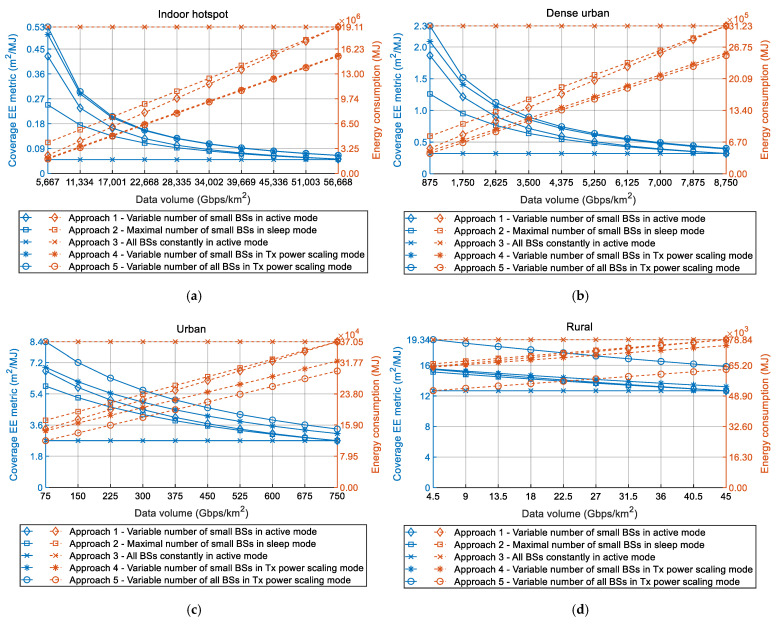
Impact of DV increase on coverage EE metrics and 5G network yearly energy consumption during the 2020s for (**a**) indoor-hotspot, (**b**) dense-urban, (**c**) urban and (**d**) rural device density class.

Since trends in the necessity of transferring increasing DVs impact both EE metrics and power consumption of 5G networks, an increase in DVs will also have an influence on the total 5G network energy consumption. In the performed analyses, the total network energy consumption refers to the energy consumption of all (small and macro) BSs located in the area of the analyzed device density class during the time period of one year. The impacts of the DV increase through 2020s on coverage EE metrics and total yearly energy consumption of 5G network for indoor-hotspot, dense-urban, urban and rural device density classes are presented in Figure 4a–d, respectively.

According to Figure 4a–d, for analyzed Approaches 1, 2, 4 and 5, an increase in DV of each device density class will impose an increase in the yearly energy consumption of the 5G network. As explained in the previous section, this is due to the fact that transferring larger DVs requires the exploitation of more BSs and BSs resources, thus resulting in the higher total instant power consumption. This consequently results in an increase of the yearly energy consumption of the analyzed device density class area (Figure 4a–d). Due to explained reasons related to lack of any deployment and RRM approach dedicated to improving EE, only in the case of Approach 3 will the total yearly energy consumption be equal to maximal energy consumption for all years in every device density class.

Although analyzed Approaches 1, 2, 4 and 5 are based on the adjustment of BSs deployment dynamics and/or operation activity with respect to the increasing DVs trend, this DV increase will have a negative impact in terms of increase of the yearly energy consumption of the 5G network. This negative impact also negatively impacts the coverage EE metrics, which will decrease with the increase of DV in all analyzed device density classes (Figure 4a–d). This is the consequence of the increase of network energy consumption when the DVs that must be transferred in the network increase. Consequently, larger energy consumption for the same size of the device density class area will, according to Equation (2), result in the degradation of the coverage EE metric.

## 8. Discussion on Performed Analyses and Obtained Results

The obtained results presented in Figure 2, Figure 3 and Figure 4 show a significant impact on the coverage and data EE metrics of DV increase in the 5G HetNets. The results presented in Figure 2 reveal the reverse influence of increased DVs on the coverage and data EE metrics. These adverse changes of data and coverage EE metrics are noticed for all approaches which use deployment and operation policies that include an adaptation of BSs resources according to DV increase. While an increase of DV in the area of analyzed device density classes has an impact on the decrease of the coverage EE metrics (Figure 2 and Figure 4), it also has an impact on the increase of the data EE metrics (Figure 2 and Figure 3). Due to such contrary changes in trends of data and coverage EE metrics, there is no optimal DV that, transferred by the network, will ensure 5G network operation with the best possible combination of data and coverage EE metrics.

To simultaneously improve both data and coverage EE metrics, the best approach is to implement 5G BSs deployment and operation strategies that have the highest level of adaptation of installed BSs resources for accommodating the increasing DV trend in any device density class. Based on the results presented in Figure 3, for any device density class, approaches that will have better data EE metric are those which have the lower instant power consumption for the same DVs that must be transferred in the same network area. Those approaches are deployment and operation Approaches 4 and 5 (Table 3), which, in comparison with Approaches 1 and 2, have lower instant power consumption for transfer of the same DVs (Figure 3). Additionally, based on the results presented in Figure 4, it can be noticed that, for any device density class, approaches that have higher coverage EE metric are also approaches that have lower yearly energy consumption for the case when the same DV must be transferred in the same area of device density class. Obviously, approaches that have the lowest energy consumption (Figure 4) will also have the lowest instant power consumption (Figure 3).

Therefore, only optimization of network instant power consumption (and consequently energy consumption) will result in simultaneous improvement of both data and coverage EE metrics. Hence, those BSs’ deployment and operation strategies that enable the best possible adaptation of BSs’ resources according to the increase of network DV will result in the optimized power consumption, which contributes to the simultaneous increase of both EE metrics. This observation further raises the necessity of implementing 5G BSs deployment and operation concepts that will ensure the transfer of constantly increasing DVs, while keeping both data and coverage 5G HetNets EE metrics at higher values as much as possible.

The further discussion concerns the values of the coverage and data EE metrics shown in Figure 2, Figure 3 and Figure 4. It is necessary to highlight that these values of EE metrics were obtained for the specific device density classes (Table 1), which consist of versatile numbers of small and macro BSs (Figure 1). Hence, every analyzed device density class has been defined in terms of the number of macro and small BSs, with the goal of satisfying the expected DV increase in the area of specific device density classes during the 2020s. However, different real-life deployments of 5G HetNets may vary in terms of the dynamics of DV increase through time and in the type and number of installed small and macro BSs. For that reason, versatile practical deployments of HetNets can differ in absolute values of coverage and data EE metrics when compared with those presented in this work. Nevertheless, the trends of the graphs presenting the impact of DV increase on data and coverage EE metrics will remain for every device density class, as with those in Figure 2, Figure 3 and Figure 4. Therefore, conclusions presented in this paper about trends in changes of data EE metric, coverage EE metric, instant power and yearly energy consumption caused by an increase in DVs can be generalized for any practical implementation of 5G network segment which belongs to a specific device density class.

An additional discussion point is related to the assumption used in the analyses according to which the DV during a single year is constant in the area of every device density class (Table 1). In reality, the instant DVs that must be transferred in the area of every device density class will vary during a day and throughout the year. To approximate these DV variations, DVs used in the analyses represent the average yearly DVs expected to be transferred by the 5G BSs located in a square kilometer area of a specific device density class. They are calculated for every year in the 2020s (Figure 2, Figure 3 and Figure 4) and for every device density class. The calculation takes into account an increase in the projected number of active user devices through the 2020s and the sum of their minimal uplink and downlink throughputs prescribed by ETSI standard for every device density class [1,2] Hence, it is reasonable to assume that the DVs used for modeling the effect of DV increase in square kilometer area of every analyzed device density class can be set to fixed DVs. For the purpose of analyses presented in this work, these fixed DVs express an average expected DVs in each year of the 2020s.

A final assumption is related to performing analyses with the excluded contribution to the total network energy consumption of other BS site elements, such as backhaul network equipment, cooling equipment and ancillary equipment. Moreover, the contribution to the overall network energy consumption of other types of BSs which can be collocated with 5G BSs at the same BSs site (e.g., 2G, 3G and 4G BSs) is not considered in the analyses. Although the energy consumption of all stated network elements contributes to the overall MN energy consumption and, therefore, impacts the network data and coverage EE metrics, this contribution was excluded from the analyses. The reason for this exclusion is in the main goal of this paper, which is dedicated to enlightening how the expected future increase in DVs will impact standardized data and coverage EE metrics of 5G networks. The analysis is, therefore, performed for 5G networks only, due to the fact that the cellular RAN has the greatest contribution to the overall MN power consumption and 5G networks will be dedicated to transferring the largest shares of DVs in the future transfer of DVs in mobile networks. For that reason, only 5G BSs and corresponding power consumption profiles were considered in the analyses. Therefore, the results of this analysis can be used for developing energy-efficient implementation and operation strategies of BSs in contemporary 5G networks.

## 9. Conclusions

The continuous increase of DVs on the global level caused by an increase in the number of mobile users and the introduction of novel and more bandwidth-demanding applications imposes challenges in deploying energy-efficient 5G networks. These challenges will additionally be contributed to by the fact that heterogeneous 5G networks must be deployed in areas of different device density classes, which will have different increases of DVs during the 2020s.

In this work, the influence of constant increase of DV during the 2020s on network EE was analyzed. The analysis was performed for different 5G network deployment and operation approaches implemented in rural, urban, urban-dense and indoor-hotspot device density classes. The two standardized EE metrics, known as data and coverage EE metrics were used for the assessment of the EE of the proposed 5G BSs deployment and operation approaches.

Obtained results show that an increase in DVs has an opposite effect on data and coverage EE metric of every device density class. This opposite trend in changes of data and coverage EE metric, caused by the increase in DV, indicates that there is no optimal amount of DVs in any of the analyzed device density classes for which a combination of both EE metrics will have the highest values. Obtained results reveal that only a reduction of 5G network instant power consumption and, consequently, energy consumption, will contribute to the simultaneous improvement of both data and coverage EE metrics. Therefore, the obtained results confirm that only those deployment and operation approaches that implement some of the RRM techniques (such as Tx power scaling and/or putting BSs in sleep mode) can bring the reduction of instant power and energy consumption, thus consequently contributing to the improvement of EE metrics of 5G RANs.

Additionally, the presented results indicate that the 5G BSs deployment and operation approaches that have the highest capabilities of adjusting BSs resources according to the increase of DV, will have the highest contribution to the improvement of both EE metrics in any device density class. It is further shown that, for any of the analyzed approaches which exploit adaptation of BSs deployment dynamics and operation activity according to increasing DV trend, the DV increase will cause an increase of the instant power consumption of the 5G network. Therefore, the analyses presented in the paper can serve as a basis in the future processes dedicated to the selection of deployment and operation strategies of 5G networks which will bring the highest network EE metrics, with respect to the permanently increasing DVs in a specific device density class.

Our future research activities will be dedicated to the investigation of the impact of DV increase on the 5G network EE of complete countries, which are composed of different in size and proportion device density classes having different DV patterns.

## Figures and Tables

**Table 1:** Parameter values for the various device density classes and analyzed BS types.

User Density Class	Average UL Throughput (Mbit/s)	Average DL Throughput (Mbit/s)	DVs for 10–100% of Max DV (Gb/km^2^)	Maximal Density of User Devices (/km^2^)	DV Percentage (%) Transmitted by Macro BSs for 10–100% of Max DV
Indoor hotspot	26.67	200	5667–56,668	250,000	0.420–0.042
Dense urban	50	300	875–8750	25,000	1.370–0.137
Urban	25	50	75–750	10,000	8.00–0.80
Rural	25	50	4.5–45	600	66.70–6.67

**Table 2:** Operating parameters of different BS types used in the analyses.

BS Parameter	5G Macro BS	5G Small BS
Spectral efficiency (bit/s/Hz/cell)	10	6
Channel bandwidth (MHz)	100	800
Number of sectors (cells)/ BS capacity (Gbit/s)	3/3	1/4.8
Average power consumption in sleep mode (W)	N/A	5
Average power consumption in active mode (W)	2000	50
Average power consumption in Tx power scaling mode (W)	1600	40

**Table 3:** Analyzed network deployment and operation approach.

	Approach 1—Variable Number of Small BSs in Active Mode	Approach 2—Maximal Number of Small BSs in Sleep Mode	Approach 3—All BSs Constantly in Active Mode	Approach 4—Variable Number of Small BSs in Tx Power Scaling Mode	Approach 5—Variable Number of All BSs in Tx Power Scaling Mode
Number of macro BSs in Tx power scaling mode	N/A	N/A	N/A	N/A	Based on DV changes
Number of small BSs in Tx power scaling mode	N/A	N/A	N/A	Based on DV changes	Based on DV changes
Number of small BSs in active mode	Based on DV changes	Based on DV changes	All	Based on DV changes	Based on DV changes
Number of small BSs in sleep mode	N/A	Maximal	N/A	N/A	N/A
Overall number of installed small BSs	Changes according to DV requirements	Maximal for satisfying full DV demand	Maximal for satisfying full DV demand	Changes according to DV requirements	Changes according to DV requirements

## Abbreviations

The following abbreviations are used in this manuscript.

2G	second-generation mobile network
3G	third-generation mobile network
3GPP	3rd Generation Partnership Project
4G	fourth-generation mobile network
5G	fifth-generation mobile network
6G	sixth-generation mobile network
BS	base station
CAGR	compound annual growth rate
DL	uplink
DV	data volume
EB	Exabyte
EC	energy consumption
EE	energy efficiency
ES	ETSI standard
ETSI	European Telecommunications Standards Institute
GHz	Gigahertz
HetNet	heterogeneous network
IoE	Internet of Everything
ITU-T	International Telecommunication Union—Telecommunications Standardization Sector
KPI	key performance indicator
mmWave	millimeter-wave
MN	mobile network
MNO	mobile network operator
mMTC	Massive Machine-Type Communications
N/A	not applicable
RAN	radio access network
RRM	radio resource management
UHD	ultrahigh-definition
UL	uplink
